# Biogeochemical Element Cycling in Plant–Soil Systems: Implications for Ecosystem Dynamics and Services

**DOI:** 10.3390/biology15020203

**Published:** 2026-01-22

**Authors:** Daniel Puppe, Wajid Zaman

**Affiliations:** 1 Leibniz Centre for Agricultural Landscape Research (ZALF), 15374 Müncheberg, Germany; 2 Department of Life Sciences, Yeungnam University, Gyeongsan 38541, Republic of Korea

Biogeochemical element cycling in plant–soil systems is fundamental for ecosystem dynamics and services [1,2]. In general, the term ‘ecosystem dynamics’ describes changes in ecosystem structures caused by interactions of organisms with the external environment in an ecosystem [3]. Ecosystem services include all ecosystem functions that are useful to humans like pollination, climate stabilization, or biomass production. In terrestrial ecosystems, plants represent the main primary producers of biomass (organic compounds) and oxygen through photosynthesis. In soils, micro- and macro-organisms recycle elements like carbon (C), nitrogen (N), and silicon (Si), maintaining soil fertility, which is essential for plant growth and corresponding ecosystem services [4,5]. Knowledge of how these organisms and plants interact is crucial to understand ecosystem dynamics and services. Nowadays, terrestrial ecosystems and their services are strongly affected by global change, which represents a grand challenge for ecosystem management and policy [6,7]. The research presented in this Special Issue seeks to address these aspects by examining the complex relationships between biogeochemical cycling, ecosystem dynamics, and the services that ecosystems provide. The 11 articles in this Special Issue provide valuable insights into how nutrient/element cycling influences ecosystem functions and how various land management practices such as afforestation or the use of fire affect microbial diversity and soil health, for example.

In two articles of this Special Issue, the impact of different agricultural practices, i.e., the use of fire [8] and Si amendment/crop straw recycling [9], on biogeochemical nutrient/element cycling in agricultural plant–soil systems was studied. Arunrat et al. [8] examined the impact of fire on soil properties and bacterial communities under rotational shifting cultivation in the tropical zone (northern Thailand). While they found positive fire effects on soil properties (e.g., increased nutrient availability), bacterial communities were negatively affected by fire (decrease in richness and diversity), but recovered relatively fast after burning, i.e., within some months. This knowledge is helpful to derive practice-related recommendations for post-fire management in traditional farming systems like rotational shifting cultivation. Puppe et al. [9] analyzed silica accumulation in potato plants and the effect of Si plant availability on long-term potato yield performance in the temperate zone (Germany). They found relatively low Si contents in the dry mass of potato leaves (up to 0.08%) and roots (up to 0.3%) and negligible Si contents in potato tuber skin and tuber flesh for plants grown in soils with different concentrations of plant-available Si (Si amendment, field experiment 1). Moreover, potato yield was not correlated to plant-available Si concentrations in soils in the long term (1965–2015, crop straw recycling, field experiment 2). Based on their results, Puppe et al. [9] ascribed the beneficial effects of Si fertilization on potato growth and yield performance reported in previous studies mainly to antifungal/osmotic effects of foliar-applied Si fertilizers and to changes in physicochemical soil properties (e.g., enhanced phosphorus (P) availability and water-holding capacity) caused by soil-applied Si fertilizers.

Other authors examined the distribution of (toxic) soil elements in areas in China, which are strongly affected by human activities. Wu et al. [10] studied the distribution of several minor and trace elements in the soil of five different forests in the core area of the Winter Olympics in the Beijing–Tianjin–Hebei region. They reported soil minor and trace elements to be mainly influenced by climatic factors and soil properties, while no direct effect of vegetation type on soil element distribution was observable. The results of Wu et al. [10] are valuable to better understand how climate change affects the distribution of minor and trace elements in soil of boreal forests in general. Xu et al. [11] analyzed soil and crop samples to assess the distribution of toxic metals (“heavy metals”) in farmlands located around smelting facilities in Jinchang City. Based on their results, they identified main areas of nickel (Ni), copper (Cu), and cobalt (Co) contamination, where Ni, Cu, and Co levels in crop samples exceeded regulatory limits. The results of Xu et al. [11] provide a valuable foundation for future establishments of remediation strategies in the investigated area.

Three studies of this Special Issue deal with N dynamics in agricultural contexts. Ahmed et al. [12] performed laboratory experiments to examine how different concentrations of the two microbial inhibitors streptomycin and cycloheximide affect nitrous oxide (N_2_O) emissions from strongly acidic soil. These authors found that high concentrations of the applied microbial inhibitors effectively reduced N_2_O emissions, while lower microbial inhibitor concentrations resulted in increased N_2_O production. The results of Ahmed et al. [12] illustrate the complexity of microbial interactions in acidic soils and highlight the importance of considering the broader ecological context when applying microbial inhibitors in agricultural plant–soil systems. Xiang et al. [13] used an automated incubation system to examine the effects of liming on N_2_O emissions from acidic soils. Their results showed that N_2_O emissions increased following liming, especially when combined with urea addition, by stimulating nitrification. These findings are useful for developing an optimal liming strategy, which alleviates soil acidity by increasing soil pH, while mitigating N_2_O oxide emissions from agricultural soils. Xu et al. [14] investigated the effects of soil N, i.e., nitrate (NO_3_^−^) and ammonium (NH_4_^+^), availability on the grass *Hemarthria altissima*. They observed that *H. altissima* specimens grown under high NH_4_^+^ levels used N mainly for carboxylation processes, while *H. altissima* specimens grown under high NO_3_^−^ levels allocated N mainly to leaf light-capturing proteins. The findings of Xu et al. [14] are useful for developing targeted N fertilization strategies to avoid over-fertilization and improve crop yields.

In three articles of this Special Issue, the results of different afforestation approaches in China are discussed in detail. Chen et al. [15] evaluated how different vegetation restoration strategies (barren land/control, disturbed short-rotation and undisturbed long-term *Eucalyptus* monocultures, a mixed native-species plantation, and a natural forest) affect soil quality and microbial communities in tropical ecosystems. While they found soil quality in undisturbed long-term *Eucalyptus* monoculture and mixed native-species plantations to be comparable to natural forest soil quality, soil microbial biomass remained lower in all plantations compared to the natural forest. The results of Chen et al. [15] represent a valuable basis for assessing the suitability of different afforestation approaches in the tropics. Du et al. [16] examined the effects of four different afforestation strategies, i.e., plantations of (i) *Salix cheilophila* and *Populus simonii*, (ii) *S. cheilophila*, (iii) *P. simonii*, and (iv) *Caragana korshinskii*, on biological soil crust properties and microbial communities in an alpine sandy land in the Gonghe Basin. Their results showed the *P. simonii* plantation to represent the most promising afforestation strategy in this area to enhance biological soil crust nutrient contents and optimize bacterial community structures. Zuo et al. [17] studied the effects of five different afforestation strategies, i.e., plantations of (i) pure *Eucalyptus*, (ii) *Eucalyptus* and *Cupressus* (2:1 ratio), (iii) *Eucalyptus* and *Cupressus* (1:1 ratio), (iv) *Eucalyptus* and *Cupressus* (1:2 ratio), and (v) pure *Cupressus*, on soil physicochemical properties, microbial communities, and metabolomes in a subtropical mountain area. They found mixed *Eucalyptus–Cupressus* plantations to represent the most promising afforestation strategy, which improved soil conditions and promoted a more diverse and functionally enriched soil microbiome, particularly at a 1:2 *Eucalyptus–Cupressus* ratio.

Last but not least, Zaman et al. [18] summarized current knowledge of biogeochemical C, N, P, sulfur (S), and Si cycles, emphasizing their roles in nutrient/element cycling, plant growth, and soil health, especially in agricultural plant–soil systems. Based on their literature review, they identified research gaps that should be filled in future studies to better understand the interlinkages between biogeochemical cycles and their responses to global change. Moreover, they called for integrated approaches that combine interdisciplinary research, technological innovation, and sustainable land-use strategies to mitigate human-induced disruptions and enhance ecosystem resilience in the face of environmental change.

The articles published in this Special Issue provide a comprehensive overview of the complex interactions that govern ecosystem functioning and offer important insights for sustainable ecosystem management in a changing world. We hope that the findings presented here will inspire future research and contribute to the development of policies and practices that promote healthier, more resilient ecosystems for the generations to come.

## Data Availability

No data was generated or analyzed during the writing of this article.
